# Development and validation of the self-regulation of blood donation scale for blood donors

**DOI:** 10.1016/j.htct.2024.09.2482

**Published:** 2024-11-07

**Authors:** Fahimeh Ranjbar Kermani, Sedigheh Amini Kafi-Abad, Mahtab Maghsudlu, Kamran Mousavi Hosseini, Fatemeh Mohammadali, Atefeh MohammadJafari

**Affiliations:** a Blood Transfusion Research Center, High Institute for Research and Education in Transfusion Medicine, Tehran, Iran; b Department of Psychiatry, Tehran University of Medical Science, Tehran, Iran

**Keywords:** Self-regulation, Motivation, Blood donation, Questionnaire, Validation, Iran

## Abstract

**Background:**

Because knowledge of blood donor motivation is crucial in guiding recruitment and retention efforts, the present study aimed at developing and validating a new scale as a multidimensional measure of blood donation motivation from the perspective of the self- determination theory.

**Methods:**

This study was conducted in three phases from September 2022 to May 2023. The first phase involved developing a draft scale based on a literature review. In the second phase, face and content validity were performed. The third phase used a cross-sectional design to assess the construct validity and internal consistency of the initial scale following administration to blood donors with a history of at least one previous whole blood donation who visited the largest blood transfusion center in Iran. Of the 420 subjects who were recruited using a mixed sampling method, 343 who fully completed the initial version of the scale were subjected to an construct validity assessment using exploratory factor analysis with Equamax rotation and internal consistency using McDonald's omega coefficient.

**Main results:**

The initial version of the scale consisted of 30 survey items with both a content validity ratio and a content validity index of 0.99. Exploratory factor analysis identified 24 items; grouped in six regulation factors (non-regulation, external regulation, introjected regulation, identified regulation, integrated regulation, and intrinsic regulation). The factors demonstrated adequate internal consistency with ω values ranging from 0.60 to 0.79.

**Conclusion:**

The present study provides psychometric support for the newly developed questionnaire to evaluate donation motivation among blood donors in Iran or in other countries with similar language, and religious and cultural values.

## Introduction

Consistent with the World Health Organization's goal of nonremunerated blood donation, in every country, voluntary donation is viewed as a foundation for a safe, sustainable blood supply.^1^

Since 2007, Iran has been one of the countries with 100 % voluntary blood donation.^2^ Recently, the World Health Organization announced that the need for blood is increasing worldwide. This need for blood is due to population growth, an increase in chronic diseases, and an increase in the number of surgeries.^3^ Because donors are the source of the blood supply, recruiting and retaining volunteer donors is of special importance in blood transfusion organizations.^4^ A valuable way to achieve this important goal is to evaluate the motivation of blood donors so that with appropriate interventions and effective methods, the goal of providing sufficient healthy blood can be achieved.

Altruistic motivations, benefits to one's health, and religious beliefs have been reported as the main motivations among Iranian blood donors.^5^ However, there is no suitable psychometric scale to assess this. In 2014, France et al. published the Blood Donor Identity Survey (BDIS), which was the first scale to assess blood donor motivation from the perspective of the self-determination theory (SDT).^6^ According to SDT, individuals can be motivated to engage in behavior by a range of external factors (i.e., the behavior is controlled by external rewards and punishments or by pressure to please significant others) and internal factors (i.e., the behavior is consistent with one's personal values).^7^ Importantly, internal motivations are seen as the most strongly related to both initiation and maintenance of behavior because they support a basic and universal psychological need for autonomy or behavioral choice.^8^, ^9^, ^10^ Concerning blood donation, it has long been suggested that as people gain experience with voluntary blood donation, they internalize a personal identity as a donor and, in so doing, are increasingly likely to engage in blood donation based on internal rather than external motivational forces.^11^

While the BDIS has demonstrated strong psychometric properties in samples of American blood donors,^12^^,^^13^ the application of this measure to Iranian blood donors is impeded by the fact that its language is English, and some of the survey items may not be generalized to the Iranian context. Further, due to differences in religion and culture, the original English scale does not have items addressing religious and cultural motivational factors that are very important in Iran.

## Objectives

Given there is no psychometric tool to investigate the motivation of blood donation among donors in Iran, the objective of the present study was to develop and validate a Self-Regulation of Blood Donation Scale (SRBDS) among Iranian blood donors. The novel scale is suitable for research on blood donation motivation.

## Methods

This study was carried out in three phases: development of the Self-regulation of Blood Donation Motivation Scale (SRBDS) in the Persian language, face and content validity, and an assessment of construct validity and internal consistency in the Persian context.

The study was approved by the Research Ethics Committee of the High Institute for Education and Research in Transfusion Medicine and was assigned the Code of Ethics IR.TMI.REC.1401.014.

## Theoretical framework

Based on the SDT, the basic regulation of human behavior is along a continuum of self-determination.^7^ This theory includes six types of motivation regulation:

• Non-regulation (amotivation) characterized by a lack of intention or action: a person does not find any meaning, value or passion to act
• Externally regulated includes behaviors that are performed to achieve external rewards
• Introjected regulation, which includes behavior performed to avoid guilt or for ego enhancement
• Identified regulation, in which the person considers the behavior to be personally important and valuable
• Integrated regulation in which the behavior is not only considered personally important, but also compatible with or part of the individual's identity
• Intrinsic motivation, which is characterized by action for the interest, pleasure, and intrinsic satisfaction resulting from the activity.

In general, more self-determination for a behavior leads to more positive outcomes and more successful self-regulation. Using the literature review, we considered the six regulatory levels in the new questionnaire (SRBDS).

Phase 1: Development of the self-regulation of blood donation scale.

The SRBDS was developed based on the self-determination theory through a literature review of articles published in English or Persian on blood donation motivation. Items related to SDT were selected.^5^^,^^6^^,^^14^, ^15^, ^16^, ^17^ After removing similar or duplicate items, 99 items were identified as the items pool in the Persian language. So, a draft of the SRBDS was prepared for the next step.

Phase 2: Face and content validity of the self-regulation of blood donation scale.

Face validity shows that the measurement tool can apparently measure the concept of the research. For the face validity of the questionnaire, the form of the questions should be logical and appropriate to the characteristics of the respondents.^18^ For face validity assessment, the item pool was evaluated based on the criteria of relevancy to the motivation of blood donation, difficulty of phrases, and clarity (lack of ambiguity) by five evaluators, including two experts in blood donation, one expert in hematology and blood banking, and two experts in neuropsychology. After deleting problematic items, the version of the SRBDS had been reduced to 78 items.

Content validity refers to the extent to which the measurement tool is representative of the entire community of questions and accurately measures different aspects of a specific concept.^18^ For content validity, the experts were asked to give their opinions regarding the wording, grammatical rules, and items relevant to any one of the six regulatory levels of the self-determination theory and determine the compliance of the scale with the theory. Based on the experts' judgment, 24 items that were deemed inappropriate or irrelevant to the self-determination theory were deleted, and 11 new items were added, resulting in a final pool of 65 items. Content validity ratio (CVR) and content validity index (CVI) were used to ensure the selection of the most important and correct items with the necessary comprehensiveness and clarity to remain in the SDT-based model. The following formula was used to determine CVR: CVR = (Ne - N/2)/(N/2), where Ne is the total number of experts who had chosen the necessity of that item, and N is the total number of experts who had commented on the statement. The average of the CVRs was considered the CVI. According to Lawshe, items, whose necessity was not confirmed by five experts, were excluded from the questionnaire,^19^ with 48 items with CVR and CVI of 0.99 remaining. Next, the 48-item questionnaire underwent further content validity analysis. The experts were asked to assign to each item one of the six regulatory styles based on self-determination theory. At this stage, 17 items that were deemed difficult or ambiguous to assign were removed, and then, the remaining 31 items assigned to the six regulatory styles were analyzed using Kendall's W correlation coefficient. The coefficient was 0.733 with a chi-squared statistic of 109.893, a degree of freedom of 30, and *p*-value <0.001, indicating an acceptable level of agreement among the experts.

Finally, to ensure that the survey items were understood by the respondents and that they could express their blood donation experience by answering the questions, the version of the SRBDS with 31 items was given to 20 blood donors visiting the main blood transfusion center in Tehran Province (the capital of Iran). They were also asked to give any suggestions about the items, such as deleting items or adding new items. After analyzing the data of the pilot study, the participants' opinions were reviewed. One item, which was deemed inappropriate by the blood donors (“I donate blood for drinks and snacks”), was removed, and the initial Persian version of the scale consisting of 30 items was established.

Phase 3: Construct validity and internal consistency of the self-regulation of blood donation scale.

## Study design

A cross-sectional design was adopted to evaluate construct validity and internal consistency of the SRBDS.

### Study subjects

This study was conducted on blood donors with a history of at least one previous whole blood donation who visited the main blood transfusion center in Tehran Province, from January to May 2023.

### Sample size

Based on Plichta et al., the required sample size to perform exploratory factor analysis (EFA) is recommended between three and ten subjects per item.^20^ In this study, the ratio of seven people per item was used. Considering the 30 items and a design effect of 1.6, the required sample size for performing EFA was estimated at 336.

### Sampling method

A mixed sampling method was used in the study. In the first step, participants were enrolled via purposive sampling. In the second step, based on the study period, 30 days as a cluster were randomly selected. The sample size was divided equally among 30 clusters. Each day cluster was classified into four groups of working hours, and the required sample size was assigned proportionally. Sampling was achieved using convenience sampling to reach the required sample size in each working hour group.

### Data and sample collection

Blood donors who declared having previous experience of blood donation were informed about the purpose of the research. Individuals who agreed to participate in the study were assured that their information would be kept completely confidential and that they could withdraw from the study at any stage. The participants were asked to answer the 30-item survey, as well as questions about demographic characteristics such as age, sex, and lifetime blood donation experience. Individuals who refused to participate were asked to answer only questions about demographic characteristics.

### Statistical analyses

For descriptive statistics, the number and frequency (%) for categorical variables and mean and standard deviation (mean ± SD).for continuous variables are reported.

Exploratory factor analysis (EFA) measures the factors that clarify the structure instruments.^21^ To check the assumption for EFA, Kaiser-Meyer-Olkin (KMO) indices above 0.8 were used to confirm the adequacy of sampling^22^ and a significant Bartlett's test of sphericity was used to confirm the adequacy of sample selection for EFA and the correlation matrix between items.^20^ Due to inter correlation between the six factors of SDT, principal component analysis (PCA) using Equamax rotation was used to test for possible factors within the survey items. The determinant criterion >0.00001 was used to confirm the absence of multicollinearity.^23^ Additional selection criteria included 1) inter-item correlations <0.6, as evidence of the absence of multicollinearity, 2) eigenvalues above 1, with the screen test to determine the number of factors,^24^ and 3) items with loading factor >0.5 in one factor. The average correlation between the items in related factors within the range of 0.15–0.50 was considered acceptable.^25^

McDonald's omega coefficient (ω) was used to evaluate internal consistency of each factor. Coefficients of 0.8–0.9 represent an excellent correlation, coefficients of 0.7–0.8 identify a good correlation, and coefficients of 0.6–0.7 indicate an acceptable correlation

An alpha error ≤0.05 was considered significant. Statistical Package for Social Sciences (SPSS) version 23 (SPSS Inc., Chicago, IL, USA) and STATA version 14 (College Station, Texas 77845 USA) software programs were used for statistical analysis.

## Results

During the study period from February to May 2023, 343 participants completed the SRBDS. Most participants were male (96.79 %) and the mean age was 40.24 ± 10.12 years.

### Construct validity and internal consistency evaluation

#### Sampling adequacy indicators in the first exploratory factor analysis

The Kaiser–Meyer-Olkin measure of sampling adequacy index was equal to 0.88, and Bartlett's test of sphericity was equal to 3453.743 (*p*-value <0.0001), which indicated the adequacy of sampling and the satisfactory status of the data to perform factor analysis.

#### Evaluation of factors

**Figure 1:**
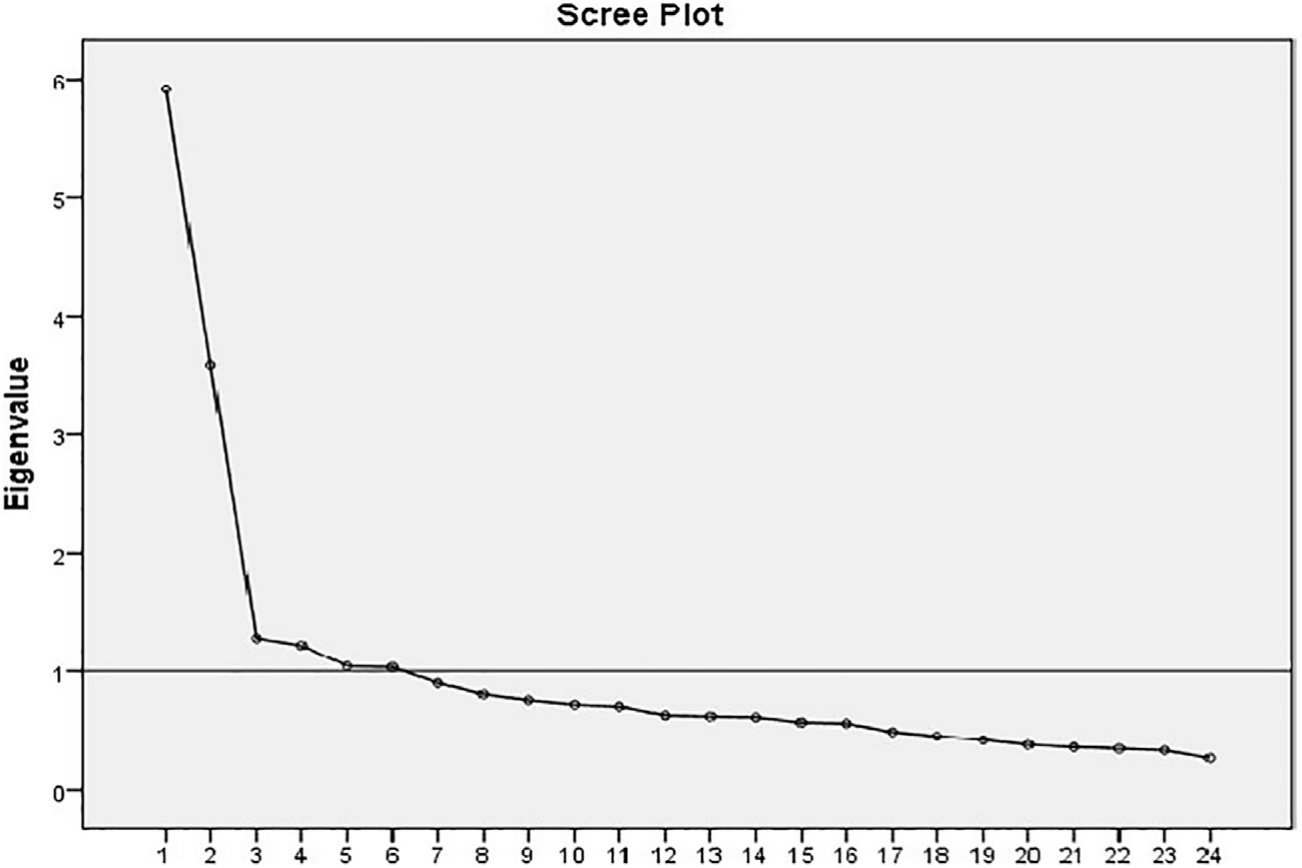
Scree plot of dimensionality of the items in six factors of the self-regulation of blood donation scale (SRBDS).

The EFA was repeated four times. In the first iteration, the 30 items were grouped into six factors. However, four items had factor loadings <0.5 and were therefore dropped, and the EFA was repeated using the remaining 26 items. This second EFA also resulted in six factors, with one item having a factor loading <0.5. This item was dropped too, and the third EFA was then conducted on 25 items, resulting in six factors; once again, a single item had a factor loading <0.5 and was then dropped. The fourth EFA included 24 items showing a six-factor structure where all items were loaded at 0.5 or higher (Table 1). The six factors accounted for 58.66 % of the total variance, including 10.67 % (factor 1), 10.35 % (factor 2), 10.09 % (factor 3), 9.88 % (factor 4), 9.61 % (factor 5), and 8.06 % (factor 6). Figure 1 shows the scree plot of the six-factor motivational model.

**Table 1:** Factor loadings and the internal consistency values of the self-regulation of blood donation scale (SRBDS).

Item	Factor					
	1	2	3	4	5	6
Non-regulation - ω* = 076						
Q2- I have no interest in donating blood	0.695					
Q8- I don't think about donating blood	0.693					
Q14- I think my blood is not suitable for others	0.617					
Q20- I have no clear feelings about donating blood	0.695					
Q29- Donating blood is not important	0.665					
External regulation - ω = 0.60						
Q6- My relatives or I have already received blood, so it is my duty to donate blood						0.625
Q11- I donate blood for free testing or physical examination						0.614
Q21- I donate blood to reduce my blood concentration						0.674
Q25- I donate blood on the recommendation of my doctor						0.661
Introjected regulation - ω = 0.76						
Q23- By donating blood, my self-confidence increases				0.576		
Q24- If I don't donate blood, I become anxious				0.732		
Q26- I feel proud to donate blood				0.675		
Q27- I feel guilty if I don't donate blood				0.702		
Identified regulation - ω = 0.76						
Q3- I donate blood to please God					0.813	
Q5- Donating blood is a religious duty					0.774	
Q15- Donating blood is a national duty					0.604	
Integrated regulation - ω = 0.75						
Q7- Donating blood is very important to me		0.642				
Q9- I am interested in donating blood		0.727				
Q10- Helping fellow human beings like donating blood is an important part of who I am		0.568				
Q13- Donating blood is consistent with my life goals		0.576				
Intrinsic regulation, ω = 0.79						
Q18- I feel happy about donating blood			0.578			
Q22- I feel satisfied by donating blood			0.691			
Q28- I think donating blood is enjoyable			0.666			
Q30- Donating blood is a good thing			0.652			

Exploratory factor analysis was conducted using extraction method of principal component analysis and rotation method of Quamax with Kaiser normalization.

ω: McDonald's omega coefficient.

Based on the definitions of the six-factor regulation model, the factors were named as follows: non-regulation, external regulation, introjected regulation, identified regulation, integrated regulation, and intrinsic regulation. As shown in Table 1, the amotivation (non-regulation) and identified regulation factors have five and three items respectively, and externally regulation, Introjected regulation, integrated regulation, and intrinsic regulation have four items each. The SRBDS in Persian and English without questions about socio-demographic characteristics are shown in Supplementary Files 1 and 2.

The internal consistency evaluation showed that the range of omega coefficient (ω) in the subscale was 0.60–0.79 (Table 1).

The average inter-item correlation showed a range of 0.266–0.496 (Table 2).

**Table 2:** Inter-item correlation in the six factors of the self-regulation of blood donation scale (SRBDS).

Factor		Minimum	Maximum	Mean
1	Non-regulation	0.290	0.459	0.385
2	Integrated regulation	0.383	0.483	0.424
3	Intrinsic regulation	0.259	0.592	0.466
4	Introjected regulation	0.3062	0.540	0.435
5	Identified regulation	0.446	0.577	0.496
6	External regulation	0.144	0.335	0.266

A moderate correlation was observed between the integrated regulation factor and the intrinsic regulation factor (*r* = 0.613). All the other correlations were below 0.6. On the other hand, there was an inverse correlation between the intrinsic regulation factor and non-regulation factor (*r* = −0.199), and between the integrated regulated factor and non-regulation factor (*r* = −0.304 - Table 3).

**Table 3:** Correlation between six factors of the self-regulation of blood donation scale (SRBDS).

Factor	Non- regulation	External regulation	Introjected regulation	Identified regulation	Integrated regulated	Intrinsic regulation
Non- regulation	1					
External Regulation	0.376	1				
Introjected regulation	0.093	0.275	1			
Identified regulation	0.029	0.201	0.503	1		
Integrated regulated	−0.304	0.040	0.414	0.460	1	
Intrinsic regulation	−0.199	0.089	0.513	0.443	0.613	1

Significant items are shown in bold and underlined. All *p*-values are <0.0001.

## Discussion

As far as we know, there was no psychometric tool to investigate the motivation of blood donation among blood donors in Iran in a multidimensional manner adapted to the country's culture. The primary objectives of the present study were to develop and validate a Self-Regulation of Blood Donation Scale (SRBDS) for Iranian blood donors.

This study used EFA with PFA to examine the motivational continuum based on SDT.^7^ The EFA identified 24 items grouped in six SDT-based factors (non-regulation, external regulation, introjected regulation, identified regulation, integrated regulation, and intrinsic regulation). The psychometric evaluation indicated good validity for the SRBDS.

In this study, five of the six factors had acceptable internal consistency or better, with the correlation coefficient ranging from 0.75 to 0.79; one factor, external regulation, was in the questionable range of internal consistency (0.60). In the BDIS, the internal consistency was acceptable or better for five factors ranging from 0.7 to 0.81 with one factor, identified regulation, being questionable with an internal consistency of 0.63.^6^ According to the context and purpose of the scale, overall, the internal consistencies were adequate.^8^

In this study, correlational analyses revealed that the factors are relatively distinct; a weak inverse correlation was observed between the factors on the opposite ends of the self-regulation spectrum (i.e., *r* = −0.199 between non-regulation and intrinsic regulation), with positive correlations noted among adjacent factors.

The present study had some notable limitations. First, the study had a cross-sectional design, so we were not able to evaluate some other aspects of the psychometric properties of the SRBDS, such as responsiveness validity. Second, due to the lack of a similar psychometric questionnaire, we could not perform some other types of validity, such as convergent validity and discriminant validity. We suggest that these validities be checked if such a questionnaire becomes available in the future.

## Conclusion

The SRBDS demonstrated adequate validity and reliability, providing initial psychometric support for the measure in the context of assessing the motivations of blood donors in Iran and other countries with a similar language and religious and cultural values. This measure may be useful in evaluating motivation regulation toward blood donation. The SRBDS can be used in developing promotional, educational, and interventional programs related to recruiting and retaining volunteer blood donors. This is important because intrinsic motivation may lead to better donor attendance and retention. Therefore, blood transfusion organizations should aim to plan strategies to create and enhance internal motivation. It seems that prospective studies are needed to determine which motivational factor is the most influential for blood donation in different provinces of the country, so that appropriate interventions can increase the level of motivation and, consequently, the rate of blood donation.

## Conflicts of interest

None.

## Appendix Supplementary materials

Image, application 1
