# Supplementary material for: Development and validation of the self-regulation of blood donation scale for blood donors
Source: Hematol Transfus Cell Ther. 2024 Nov 7;46(Suppl 6):S299–305. doi: 10.1016/j.htct.2024.09.2482 (PMC11726103; doi:10.1016/j.htct.2024.09.2482)
Supplement: Supplementary file 1 [file mmc1.docx]

**Supplementary file 1:**

**پرسشنامه خود تنظیمی اهدای خون در اهداکنندگان خون**

| ب- لطفا برای هریک از عبارات زیر گزینه ای را انتخاب نمایید که به درستی نظر شما را نسبت به اهدای خون نشان دهد. | کاملا درست | درست | تا حدی درست | نادرست | کاملا نادرست |
| --- | --- | --- | --- | --- | --- |
| 1. من هیچ علاقه ای به اهدای خون ندارم. |  |  |  |  |  |
| 1. برای رضایت خدا خون اهدا می کنم. |  |  |  |  |  |
| 1. اهدای خون یک وظیفه مذهبی است . |  |  |  |  |  |
| 1. خودم یا بستگانم قبلا خون دریافت کرده ایم پس وظیفه من است که خون اهدا کنم. |  |  |  |  |  |
| 1. اهدای خون برای من بسیار مهم است. |  |  |  |  |  |
| 1. من به اهدای خون فکر نمی کنم. |  |  |  |  |  |
| 1. من به اهدای خون علاقه دارم. |  |  |  |  |  |
| 1. کمک به همنوع مثل اهدای خون بخش مهمی از شخصیت من است. |  |  |  |  |  |
| 1. من برای انجام بعضی آزمایشات و یا معاینه جسمی رایگان خون اهدا می کنم. |  |  |  |  |  |
| 1. اهدای خون با اهداف زندگی من همخوانی دارد. |  |  |  |  |  |
| 1. فکر می کنم خون من برای دیگران مناسب نیست. |  |  |  |  |  |
| 1. اهدای خون یک وظیفه ملی است. |  |  |  |  |  |
| 1. با اهدای خون احساس شادی می کنم. |  |  |  |  |  |
| 1. هیچ احساس روشنی نسبت به اهدای خون ندارم . |  |  |  |  |  |
| 1. برای کاهش غلظت خونم، خون اهدا می کنم . |  |  |  |  |  |
| 1. با اهدای خون احساس رضایت می کنم. |  |  |  |  |  |
| 1. با اهدای خون، اعتماد به نفسم بالا می رود. |  |  |  |  |  |
| 1. اگر خون اهدا نکنم دچار اضطراب می شوم . |  |  |  |  |  |
| 1. به توصیه پزشک معالجم خون اهدا می کنم. |  |  |  |  |  |
| 1. با اهدای خون احساس غرور می کنم.. |  |  |  |  |  |
| 1. اگر اهدای خون نکنم احساس گناه می کنم . |  |  |  |  |  |
| 1. به نظر من اهدای خون لذت بخش است. |  |  |  |  |  |
| 1. اهدای خون اهمیتی ندارد. |  |  |  |  |  |
| 1. اهدای خون یک کار خوب است. |  |  |  |  |  |
| عامل 1) بیانگیزگی = گزاره های 1، 6، 11، 14 و 23، عامل 2 (انگیزه بیرونی تنظیم شده = گزاره های 4، 9، 15 و 19 ، عامل 3) انگیزه بیرونی درونی تنظیم شده = گزاره های 17، 18، 20 و 21 ، عامل 4 (انگیزه بیرونی همانندسازی شده = گزاره های 2، 3 و 12 ، عامل 5 (انگیزه بیرونی یکپارچه تنظیم شده = گزاره های 5، 7، 8 و 10 و عامل 6 (انگیزه درونی = گزاره های 13، 16، 22 و 24 | | | | | |

**Supplementary file 2:**

Blood donation self-regulation questionnaire for blood donors

| Please choose the option that correctly shows your opinion about blood donation for each of the following statements. | Very true | True to a Great Extent | Somewhat true | Untrue to a Great Extent | Not true at all |
| --- | --- | --- | --- | --- | --- |
| 1 I have no interest in donating blood. |  |  |  |  |  |
| 2 I donate blood to please God. |  |  |  |  |  |
| 3 Donating blood is a religious duty. |  |  |  |  |  |
| 4 My relatives or I have already received blood, so it is my duty to donate blood. |  |  |  |  |  |
| 5 Donating blood is very important to me. |  |  |  |  |  |
| 6 I don't think about donating blood. |  |  |  |  |  |
| 7 I am interested in donating blood. |  |  |  |  |  |
| 8 Helping fellow human beings like donating blood is an important part of who I am. |  |  |  |  |  |
| 9 I donate blood for free for some tests or physical examinations. |  |  |  |  |  |
| 10 Donating blood is consistent with my life goals. |  |  |  |  |  |
| 11 I think my blood is not suitable for others. |  |  |  |  |  |
| 12 Donating blood is a national duty. |  |  |  |  |  |
| 13 I feel happy by donating blood. |  |  |  |  |  |
| 14 I have no clear feelings about donating blood. |  |  |  |  |  |
| 15 I donate blood to reduce my blood concentration. |  |  |  |  |  |
| 16 I feel satisfied by donating blood. |  |  |  |  |  |
| 17 By donating blood, my self-confidence increases. |  |  |  |  |  |
| 18 If I don't donate blood, I get anxiety. |  |  |  |  |  |
| 19 I donate blood on the recommendation of my doctor. |  |  |  |  |  |
| 20 I feel proud to donate blood. |  |  |  |  |  |
| 21 I feel guilty if I don't donate blood. |  |  |  |  |  |
| 22 I think donating blood is fun. |  |  |  |  |  |
| 23 Donating blood is not important |  |  |  |  |  |
| 24 Donating blood is a good thing. |  |  |  |  |  |
| Factor 1) Amotivation = Q1 + Q11 + Q14+ Q23; Factor 2) External regulation = Q4 + Q9 + Q15+ Q19; Factor 3) Introjected regulation = Q17 + Q18 + Q20+ Q21; Factor 4) Identified regulation = Q2 + Q3 + Q12; Factor 5) Integrated regulation = Q5 + Q7 + Q8+ Q10; Factor 6) Intrinsic regulation = Q13 + Q16 + Q22+ Q24 | | | | | |
